# Supplementary material for: Spatial variation and determinants of solid fuel use in Ethiopia; Mixed effect and spatial analysis using 2019 Ethiopia Mini Demographic and Health Survey dataset
Source: PLoS One. 2023 Nov 30;18(11):e0294841. doi: 10.1371/journal.pone.0294841 (PMC10688864; doi:10.1371/journal.pone.0294841)
Supplement: Attachment — Submitted filename: Response to Editor.docx [file pone.0294841.s002.docx]

**Response to Academic Editor**

**Dear; Editor and Reviewers,** Please accept our revised manuscript and note our point-by point response to Academic editor and reviewers below for the manuscript titled **“Spatial variation and determinants of solid fuel use in Ethiopia; Mixed Effect and spatial analysis using 2019 Ethiopia Mini Demographic and Health Survey dataset” ,,with Manuscript ID. PONE-D-23-14224**”

1. Thank you for updating your data availability statement. You note that your data are available within the Supporting Information files, but no such files have been included with your submission. At this time we ask that you please upload your minimal data set as a Supporting Information file, or to a public repository such as Figshare or Dryad. Please also ensure that when you upload your file you include separate captions for your supplementary files at the end of your manuscript.

As soon as you confirm the location of the data underlying your findings, we will be able to proceed with the review of your submission.

**Authors’ response:** We thank you for this comment, we have included the figure which indicate as it generated for ArcGIS version 10.7.1 at each map by saying “Own map output from ArcGIS version 10.7 analysis.”
